# Long Recording Sequences: How to Track the Intra-Individual Variability of Acoustic Signals

**DOI:** 10.1371/journal.pone.0123828

**Published:** 2015-05-13

**Authors:** Thierry Lengagne, Doris Gomez, Rémy Josserand, Yann Voituron

**Affiliations:** 1 Université de Lyon, UMR5023 Ecologie des Hydrosystèmes Naturels et Anthropisés, Université Lyon 1, ENTPE, CNRS, 6 rue Raphaël Dubois, 69622, Villeurbanne, France; 2 UMR 7179 CNRS National Museum of Natural History, Brunoy, France; University of Tours, FRANCE

## Abstract

Recently developed acoustic technologies - like automatic recording units - allow the recording of long sequences in natural environments. These devices are used for biodiversity survey but they could also help researchers to estimate global signal variability at various (individual, population, species) scales. While sexually-selected signals are expected to show a low intra-individual variability at relatively short time scale, this variability has never been estimated so far. Yet, measuring signal variability in controlled conditions should prove useful to understand sexual selection processes and should help design acoustic sampling schedules and to analyse long call recordings. We here use the overall call production of 36 male treefrogs (*Hyla arborea*) during one night to evaluate within-individual variability in call dominant frequency and to test the efficiency of different sampling methods at capturing such variability. Our results confirm that using low number of calls underestimates call dominant frequency variation of about 35% in the tree frog and suggest that the assessment of this variability is better by using 2 or 3 short and well-distributed records than by using samples made of consecutive calls. Hence, 3 well-distributed 2-minutes records (beginning, middle and end of the calling period) are sufficient to capture on average all the nightly variability, whereas a sample of 10 000 consecutive calls captures only 86% of it. From a biological point of view, the call dominant frequency variability observed in *H*. *arborea* (116Hz on average but up to 470 Hz of variability during the course of the night for one male) challenge about its reliability in mate quality assessment. Automatic acoustic recording units will provide long call sequences in the near future and it will be then possible to confirm such results on large samples recorded in more complex field conditions.

## Introduction

During the last four decades, acoustic communication has been extensively studied to understand the behavioural processes associated to sound emission and reception. Using manipulated signals, many studies have determined how environmental constraints influence signal design and evolution. They have identified the relevant acoustic features and their functions in key behavioural processes like species/individual recognition or mate choice [1]–[3], and the trade-off between natural and sexual selection [4], [5]. Many behavioural contexts assume a low intra-individual variation over short temporal scales (days or weeks) for acoustic features involved in individual signature [6] or in honest signalling of individual quality [4]. Yet, this theoretical assumption has so far been supported by short recordings, since long acoustic sequences have long been difficult to acquire and tedious to analyse. These limitations are overcome by recently developed automatic sensors which allow recording long acoustic sequences in natural environments, mainly used for biodiversity survey or endangered species monitoring [7], [8], and by the automation of signal analysis and classification, which facilitates the exploitation of long sequences [9], [10].

Hence, in the near future, these technologies should allow estimating intra-individual variability based on several thousand calls emitted in field conditions. Such estimation will be necessary controlled for many abiotic and biotic parameters known to influence acoustic signals such as wind, rain, temperature, biotic noise, social context, emitter size and energetic level [11]–[16] and reference to long recordings made in controlled conditions will help to understand field results. Surprisingly, to date, there is not a single study focusing on intra-individual variability based on several hours recordings performed in controlled laboratory conditions.

The study of call variability is particularly acute in the sexual selection process. Since direct mate quality assessment is often impossible, females should rely on male signals to estimate their quality and to gain direct or indirect benefits. Call dominant frequency (DF)—frequency with the highest energy—is one of the signal characteristics most studied to date in many groups (insects: [17], mammals: [18], birds: [19] and anurans: [20]. In anurans, DF is usually considered as an index signal as many empirical studies show a negative correlation between call DF and individual mass, both at interspecific (e.g. in 116 Australian species, [21] and intraspecific level (reviews in [22], [23]). From a mechanistic point of view, DF depends on the frequency at which vocal cords vibrate, heavier vocal cords vibrating at lower frequency [24]. Vocal cords mass is generally positively linked to individual mass, at both inter and intraspecific level. Larger males are more likely to be of greater quality (better abilities to survive, to outcompete with other males, to fertilize eggs [25], [26]). Hence, DF is often considered an honest signal. To date, almost all of the studies investigating the within-individual variability in dominant frequency have shown a low coefficient of variation (CV, Table 1), thus classifying this call property as a static one in contrast to dynamic properties like call duration or call rate [27]. However, these studies are often based on very small call samples relative to the 15,000 calls a male can emit during a single calling night (see [27]-[39], Table 1).

**Table 1:** Within-male variations in dominant frequency in several anurans.

Species	Mean DF (Hz)	Mean within-Male CV (%)	sample	reference
*Agalychnis moreletti*	1233	8.3^e^	from 4 to 68cc	[28]
*Allobates femoralis*	3426^a^	2.86±1.77^f^	from 3 x 5cc	[29]
*Bufo americanus*	1795^b^	1.3^md^ ^,^ ^e^ 3.2^md^ ^,^ ^g^	from 2 to 8cc	[30]
*Bufo viridis*	˷1400^C^	1.96±1.38^b^ ^,^ ^e^ 3.3±2.6^f^	from 3 pulses/call from 1 bout	[31]
*Hyla arborea*	2139	0.91±0.39^e^ 2.96±2.27^f^	from 1 to 26 bouts^h^	[32]
*Hyla arborea*	2121	2.7±1.6^e^	from 27 distributed calls^i^	[33]
*Hyla ebraccata*	3256	1.1^e^	from 2 to 3 calls	[34]
*Hyla intermedia*	2520	2.2^e^	from 42 to 641 cc (3–14 bouts)	[35]
*Hyla versicolor*	2232	0.8^e^	from 5 calls (1 bout)	[27]
*Odorrana tormota*	6530^d^	13.1±2.8^f^	from 16 to 90 cc	[36]
*Physalaemus enesefae*	898	1.92^e^	from 5 to 10 cc	[37]
*Rana catesbeiana*	219	1.5^e^	from 19 to 20 cc	[38]
***Rana clamitans***	**393**	**2.04** ^e^	**from 8 to 10 cc**	**[39]**

DF: dominant frequency; cc: consecutive calls

^a^: call mid-frequency instead of DF

^b^: weighted average on several values given for different years or different populations

^c^: fundamental frequency instead of DF

^d^: mean fundamental frequency on frequency modulated long calls

^e^: within the same record

^f^: between different records from different nights

^md^: median instead of mean

^g^: between different records

^h^: about 20 calls/bout; DF determined by averaging spectral properties over a complete bout

^i^: 3 calls at the start, 3 at the middle and 3 at the end of the bout, on 3 bouts from the start, the middle and the end of the record

We anticipate that a better description of variability in signal production will foster a reappraisal of the function and evolution of acoustic signals, and help design efficient automated methods to analyse long recordings. Here, we investigated within-male variability in dominant frequency at night scale in the European treefrog (*Hyla arborea*). This well-studied lek-mating hylid species shows a negative correlation between mass and dominant frequency [32], [33], [40], [41]. Moreover, in this species an experimental study shows a female preference for low call frequency: males calling with a frequency content 190 Hz lower than the average value of the population are more attractive for females [41]. In controlled laboratory conditions, we set out (1) to explore temporal patterns of variation in dominant frequency at individual scale, and (2) to test the efficiency of different sampling methods at capturing this variability with a reasonable sampling effort to offer a convenient tool for future studies on animal calls.

## Materials and Methods

### Capture, housing conditions and ethic statement

Individuals came from a French population located near Lyon, called ‘Planches’ on Crémieu plateau (5°21'7"E, 45°44'20"N). During the breeding season (April and May 2009), 36 males were captured individually by hand when calling at the pond (12 males by night during 3 nights). They were then transported by car to the housing room of our laboratory and immediately placed in individual terraria (25x17x15cm) with a water-filled basin and a tree branch. Males were fed *ad libitum* with domestic crickets (*Acheta domesticus*) during 3 to 4 days for acclimation, before the experiment begun. Males were exposed to natural photoperiod and chorus noise was broadcast each night from 08:00pm to 02:00am. Temperature was kept at 22 ± 1°C. After acclimation, males were recorded individually during a whole night. At the end of the record, males were weighed and measured (femur-tibia length).

### Ethic statement

This study was conducted in accordance with French laws and with the approval of the Préfecture de l’Isère (decision 2007–03328), the Direction of Veterinary Services (permit DSV no. 69266347) and University Lyon1 Ethic Committee (decision BH201116). After recordings all males were fed ad libitum for 2 days. The night following these 2 days, they were all released at the capture site.

### Recording

Twelve males were recorded simultaneously during one night. Each terrarium was placed in an individual semi-anechoic chamber the day of the recording at 08:00pm. Terraria had plastic mesh sides and lid instead of glass sides in order to avoid sound reflectance. Calls were recorded with a Sony ECM-T6 microphone placed in each chamber and plugged in a Roland R-44 4-channels recorder or a Tascam US 144 soundcard connected to a notebook computer (Fs: 44.1KHz, 16 bits). Each semi-anechoic chamber was open on the front side to avoid stressing males. Males were stimulated by broadcasting a repeated 2-minutes chorus noise, via an amplified loudspeaker (KH pas-100) connected to a CD player and placed 3m in front of the chambers. This stimulating record was a record in which no male was louder than the others in order to avoid any risk of the tested male responding to the calls of a particular competitor [3]. Although recordings chambers were open on their front side, they were not placed face to face. As a consequence, sounds perceived by a focal male from the other adjacent males were very weak compared to the volume of the stimulating records that were broadcasted. Indeed, the signal-to-noise ratio of the neighbouring male was -9 dB below the background noise and this value remain the same during the whole night due to the constant amplitude value of the chorus noise broadcast by loudspeaker. Hence, we considered that males were physically and acoustically isolated from one another during our experiment.

### Measure of dominant frequency variability

**Fig 1:**
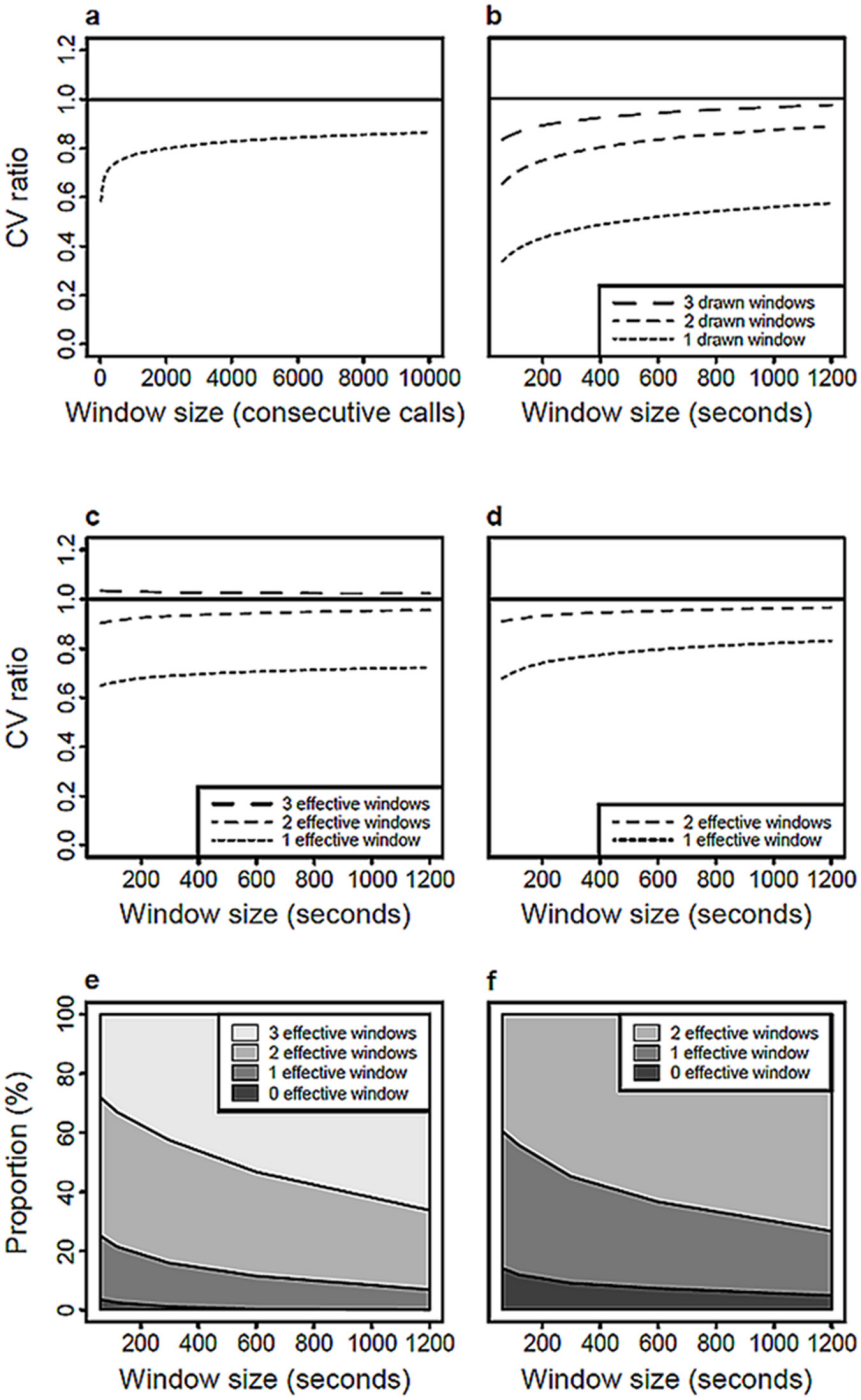
Mean effect of window size on the CV ratio (estimated CV / total CV); a using method A (sampling consecutive calls), b using method B (sampling one or several temporal window(s)). Methods B with 3 and 2 drawn windows are respectively refines in **c** and **d** by taking into account the number of effective windows (drawn windows containing calls). The proportions of effective windows are shown for these two methods respectively in **e** and **f**. The solid lines on **a, b, c** and **d** represent perfect estimations of the nightly variability.

We extracted dominant frequency for each call of the entire night recording, using Avisoft Saslab software. Call dominant frequency was here defined as the frequency with the highest energy, in a frequency range between 1.8 and 3 kHz (Fig 1). To calculate it, mean power spectra were automatically computed for each call with a Fast Fourier Transform of 256 points and a 16 kHz sampling rate. These parameters yielded frequency values with an accuracy of 62.5Hz. Such a value corresponds to about 2.5% of the mean dominant frequency value in our species. Note that the between male CV calculated in *H*. *arborea* for dominant frequency is about from 5.14% to 8.20% [32], [33]. Also note that in *Hyla ebraccata*, the hylid with the most accurate perception for which data is available in literature, female discrimination threshold for dominant frequency is about 8.6% [34]. For each male, mean, standard deviation (SD) and CV of dominant frequency were calculated on the entire night.

### Test of different sampling methods to assess frequency variability

We developed a program SONIO (http://sites.google.com/site/sonioprogram/) to test different sampling methods on Avisoft outputs. Each method was defined by two parameters: the number of subsamples to randomly draw from the entire night record and the subsample window size (in consecutive calls or in seconds). Once the method defined, the program randomly drew the subsamples and computed the mean value of dominant frequency, its estimated SD and estimated CV value. This sampling was repeated 1,000 times and the program computed a CV ratio (estimated CV value / total CV computed over the entire night) representing the fraction of the total variability captured. The sampling methods mimicked different possible decisions of an experimenter deciding *a priori* to adapt or not the record duration to male calling activity.

First, we defined windows by their number of consecutive calls (Method A). We explored 10 different window sizes (10, 20, 50, 100, 200, 500, 1000, 2000, 5000 and 10 000 consecutive calls). The smallest value represented a sample close to those often used in the literature (Table 1). Whenever the window size exceeded the total number of calls emitted by a male, we took a CV ratio of 100% as the sample captured the overall variability.

Second, we defined windows by their duration in seconds (Method B). We explored 3 cases in which respectively 1, 2 or 3 temporal windows were randomly chosen from the entire night. We constrained the program to choose windows discontinuous in time. When 2 windows (3 windows) were chosen, the total time a male called was divided in 3 parts (5 parts) and the 2 windows (3 windows) were chosen in the 1st and 3rd parts (1st, 3rd, and 5th parts). For each case, we explored 5 window sizes of 1, 2, 5, 10 and 20 minutes. For cases with several temporal windows, the CV value was computed over all calls of all windows taken together. Since windows were drawn regardless of the number of calls, some windows were without call. We thus computed the number of effective windows (with calls), which ranged from 0 to the number of temporal windows. We took this parameter into account in the analysis.

### Statistical analysis

We explored the variations of CV ratio using linear mixed models suited to repeated observations on the same individuals [42]. All models included individual as random effect (on both the intercept and the slope). We tested window size as fixed effect. Since the relationship between window size and CV ratio appeared to be log-linear, we computed the natural logarithm of window size in the models. Coefficients and standard errors (SE) were computed using a restricted maximum likelihood approach. Factor significance was tested using Wald z tests [42]. Likewise, correlations between dominant frequency and other call properties were investigated using linear mixed models included individual as a random factor. Correlations between total CV and individual characteristics were investigated using linear models. All statistics were performed using R 2.11.1 (R Development Core Team 2010) with the package ‘nlme’ for linear mixed models [43]. For all models, we checked for variance homogeneity, residual normality and independence.

## Results

The males emitted on average 14 546 ± 8644 calls (mean ± SD; range: 561–31 641 calls). Mean dominant frequency was on average 2524 ± 141 Hz (mean ± SD; range: 2250–2803 Hz). Within-individual CVs computed on dominant frequency measures from the entire night were 4.57 ± 2.00% (mean ± SD; range: 1.94–10.29%).

When using consecutive calls (method A), the CV ratio increased with the logarithm of window size, resulting in a better estimation of the total CV (Table 2, Fig 1a). A sampling of 10 000 consecutive calls captured on average up to 86% of the nightly variability in dominant frequency. Note that this percentage is influence by the fact that 11 of the 36 males have emitted less than 10 000 calls during the night; hence such a sample captured 100% of the nightly variability for these males.

**Table 2:** Effect of window size on the CV ratio (estimated CV / total CV) using the different sampling methods.

		Intercept		Log (window size)	
		fixed effect	Random effect	fixed effect	Random effect
	Windows (eff.)	estimate ± se	sd	estimate ± se	sd
Method A	1 (1)	0.492 ± 0.034***	0.207	0.040 ± 0.005***	0.029
Method B	1 (0 or 1)	0.013 ± 0.056	0.337	0.079 ± 0.009***	0.054
	2 (from 0 to 2)	0.336 ± 0.083***	0.499	0.078 ± 0.012***	0.069
	2 (2)	0.832 ± 0.053***	0.311	0.019 ± 0.009*	0.052
	2 (1)	0.471 ± 0.057***	0.327	0.051 ± 0.011***	0.061
	3 (from 0 to 3)	0.639 ± 0.053***	0.315	0.047 ± 0.008***	0.047
	3 (3)	1.048 ± 0.060***	0.347	-0.004 ± 0.008	0.047
	3 (2)	0.829 ± 0.044***	0.258	0.018 ± 0.006**	0.034
	3 (1)	0.546 ± 0.081***	0.351	0.025 ± 0.016	0.061

Individual is here considered as a random factor acting both on the intercept and the slope. For method A, windows size is considered in consecutive calls whereas for methods B, it is considered in seconds. Eff. = effective windows;

*: p<0.05;

**: p<0.01;

***:p<0.001

Using discontinuous time windows (method B) yielded a better estimate of nightly variability than using a continuous window (Table 2, Fig 1b). A total of 3 distinct 10-minutes time windows captured 94% of the nightly variability. Increasing window size to 20 minutes captured an additional 3% of the nightly variability. By contrast, decreasing the number of windows resulted in an underestimation of dominant frequency variability. For instance, 2 distinct 10-minutes windows captured 83% of the total variability while one 10-minutes window only captured 52% of the total variability (Table 2, Fig 1b). In fact, when considering the number of effective windows, two 10-minutes windows captured 95% of the total variability (Table 2, Fig 1c and 1d) whereas three 10-minutes windows captured all the variability whatever the window size (Table 2, Fig 1c). Finally, the effect of window size is due to the fact that the probability of getting a window without call is negatively correlated to window size. For instance, the probability of getting 3 effective windows is greater for a window size of 20 minutes compared to a window size of 2 minutes (Fig 1e and 1f).

## Discussion

### Nightly frequency variability in H. arborea

At night scale, total CV found in this study are greater than values generally observed in the literature for dominant frequency (table 1), even if this variability never achieves values of variability found for dynamic call properties (see results concerning call parameters of three species Table1 in [27]). By considering subsamples of 10 consecutive calls, values are in agreement with most of those observed in the literature. Yet, this method achieves capturing only 60% of the nightly variability in dominant frequency, a clear underestimation of intra-individual variability. In the European treefrog, this variability seems to occur at large temporal scale, mainly with sudden discontinuities undetected by small samples. Such variations are unlikely due to changes in environmental factors such as temperature (kept constant in our experiment) or acoustic stimulation (the stimulating record was looped and males were acoustically isolated from each other). Moreover, patterns of temporal variability differ between males.

Dominant frequency variability assessed through one record (from 1 to 26 call bouts, table1, [32]) is very low compared to values we obtained on small samples. This is probably be due to a crucial methodological difference between the two studies: in the present study dominant frequency was measured for each call whereas, in Friedl & Klump’s study, the dominant frequency was measured by averaging spectral properties over a complete call bout, thus annihilating the intra-bout variability [32]. Nevertheless, their study also investigates variability of dominant frequency at a larger scale by considering measures made on different nights. Hence, they found CV from 0 to 9.63% (after correction for temperature and number of male calling), a range of values closer to those we observed on one entire night, suggesting that a part of variability cannot be assessed by small samples.

### Dominant frequency: a honest acoustic parameter?

Given that female choice and male decisions during contests are based on very small samples (e.g. for female choice: [44]; for male contests: [45]; for sneaker strategy: [46]) the sudden discontinuities observed in the present study may lead to a poor estimation of male quality through dominant frequency. This estimation may be different depending on time at which the congener receives the signal raising the issue of the honesty of this call property. On average, we measured a DF variability of 116 Hz in our dataset but the difference during the course of the night can be as strong as 470 Hz. Can dominant frequency still be considered as an index signal (sensu Maynard-Smith and Harper [47])? In the literature, dominant frequency is usually considered an index signal as many empirical studies have shown a negative correlation between call dominant frequency and individual mass, both at interspecific [21] and intraspecific level (reviews in [22], [23]). It is also true in Hyla arborea despite the short duration of samples used [32], [41]. Is it costly for a male to shift his dominant frequency? In other words, can we consider call dominant frequency as a cost-added signal? It is a plausible possibility. In anurans, there is a mismatch between the small size of radiating structures (mainly the vocal sac) and the principal wavelengths of the call; thereby, lowering call dominant frequency supposes lowering efficiency in converting metabolic energy into acoustic energy during call production [48]. Hence, all other properties being equal, it should be costlier for a male to produce lower frequency calls (supported by [49]).

Biomechanical processes involved in sound production and more particularly the possible costs associated to modification of call dominant frequency need to be more accurately studied to determine whether within-male variability is due to passive or active shifts in call characteristics. For instance, variability of dominant frequency observed at large scale may be due to a greater variability within call bouts at the beginning and/or at the end of the night, due to a ‘warm-up’ or ‘exhaustion’ effects respectively.

Concerning mate choice, many other parameters than frequency are used by female to determine male quality in this species such as call timing [3] call amplitude as well as within bout call rate [41], [50]. However, once again, the variability of these components during the night remain unknown. At last *H*. *arborea* is one of the few species for which females use coloration of male vocal sac to determine male quality [51]–[53]. Contrary to DF, we can hypothesize that such coloration is stable at a night scale although it is known that important variations are possible within a short time delay [54].

### How to investigate dominant frequency variability?

To get a better assessment of dominant frequency variability over the night, a first solution can consist in increasing the number of consecutive calls recorded. Yet, the window size required to obtain a good estimate of total CV is huge and unrealistic to implement (even 10 000 consecutive calls hardly captures 86% of the total CV). Increasing the number of consecutive calls recorded is inefficient at capturing intra-individual variability in dominant frequency. Therefore, instead of increasing the number of consecutive calls to be analysed, we advocate to use records made at different times during the night.

Depending on recording conditions, two sampling strategies can be adopted. Firstly, when records are made in the field in a large population with many calling males, it should be easy to find calling males and thus to avoid the risk of ‘recording’ a silent male. The experimenter should decrease window size and maximize the number of samples—two or three—recorded for each male. For instance, in the European treefrog, two 2-minutes effective windows largely separated in time capture 92% of the variability and three 2-minutes effective windows are sufficient to capture all the variability. Alternatively, when records are made on a small number of individuals (small population or small number of males to record), the experimenter should optimize the number of males correctly recorded. The experimenter should thus increase window size to reduce the probability of recording males during a silent period. Since this probability is not negligible, we suggest to draw 3 temporal windows evenly distributed in time and to maximize the duration of these windows to optimize the probability of getting at least 2 effective windows for each male. For instance, in our case, by drawing randomly three 10-minutes windows, an experimenter estimates in average 94% of the variability in call dominant frequency. Note that similar methodological efforts have already been made by Castellano et al. [33]. They sample calls over an entire recording. Yet, they find a variability in DF similar to what we capture by analysing 10 consecutive calls. Although their animals may have been poorly variable, the obtained values may more likely reflect that recordings were too short compared to the duration of a calling night.

## Conclusion

This study (i) clearly shows that the call dominant frequency variability is dependent upon the way to record calling and (ii) strongly suggests that this parameter is historically underestimated in literature. The DF variability measured in our study challenges the physical link between signal characteristic and emitter quality involved in the definition of index signal [47]. This physical link appears to be looser than previously expected and its reliability in mate quality assessment should be questioned. From a methodological point of view, our results underline that various methods of analysis differ in their efficiency at capturing variability in acoustic parameters. These results should help for the design of recording schedules that could be used for automatic recording units in the field. Alternatively, analysing only a number of relevant subsamples should prove an efficient way to exploit long recordings and assess the variability of a large range of acoustic parameters in various field conditions or in different taxa such as birds or insects.
